# Update on a Pharmacokinetic-Centric Alternative Tier II Program for MMT—Part II: Physiologically Based Pharmacokinetic Modeling and Manganese Risk Assessment

**DOI:** 10.1155/2012/791431

**Published:** 2012-05-07

**Authors:** Michael D. Taylor, Harvey J. Clewell, Melvin E. Andersen, Jeffry D. Schroeter, Miyoung Yoon, Athena M. Keene, David C. Dorman

**Affiliations:** ^1^Health, Safety, Environment, and Security, Afton Chemical Corp., Richmond, VA 23219, USA; ^2^Institute for Chemical Safety Sciences, The Hamner Institutes for Health Sciences, Research Triangle Park, NC 27709, USA; ^3^College of Veterinary Medicine, North Carolina State University, Raleigh, NC 27606, USA

## Abstract

Recently, a variety of physiologically based pharmacokinetic (PBPK) models have been developed for the essential element manganese. This paper reviews the development of PBPK models (e.g., adult, pregnant, lactating, and neonatal rats, nonhuman primates, and adult, pregnant, lactating, and neonatal humans) and relevant risk assessment applications. Each PBPK model incorporates critical features including dose-dependent saturable tissue capacities and asymmetrical diffusional flux of manganese into brain and other tissues. Varied influx and efflux diffusion rate and binding constants for different brain regions account for the differential increases in regional brain manganese concentrations observed experimentally. We also present novel PBPK simulations to predict manganese tissue concentrations in fetal, neonatal, pregnant, or aged individuals, as well as individuals with liver disease or chronic manganese inhalation. The results of these simulations could help guide risk assessors in the application of uncertainty factors as they establish exposure guidelines for the general public or workers.

## 1. Introduction

As an essential element, manganese (Mn) is required for normal function of the central nervous system (CNS) and other tissues [1]. As with all other metals, manganese toxicity can occur with excessive exposure. A variety of clinical effects are associated with manganese toxicity, including manganism, a parkinsonian movement disorder that primarily affects dopaminergic and *γ*-aminobutyric acid- (GABA-) containing mid-brain structures that control motor functions [2]. More subtle effects can also occur. For example, workers exposed chronically to manganese can develop changes in visual reaction time, hand steadiness, and eye-hand coordination [3]. These neurotoxic syndromes develop when either manganese intake is excessive (e.g., following high-dose oral, inhalation, or parenteral manganese exposure) or when hepatobiliary clearance of this metal is impaired. This observation suggests that the dose of manganese delivered to target regions within the CNS is the primary determinant for manganese neurotoxicity.

The U.S. Environmental Protection Agency's (USEPA) list of hazardous air pollutants includes manganese compounds. The USEPA and health agencies in other countries have raised concerns that chronic inhalation of low levels of manganese in ambient air may pose a risk to public health due to the possible accumulation of manganese in target tissues [4]. These concerns prompted the USEPA to call for a series of pharmacokinetic studies, as well as the development of physiologically based pharmacokinetic (PBPK) models for manganese as part of the testing requirements for the organometallic fuel additive methylcyclopentadienyl manganese tricarbonyl (MMT^®^, a registered trademark of Afton Chemical Corporation) [5]. Part I of this two part series discussed the development of the USEPA's Alternative Tier II testing program for MMT that collected critical pharmacokinetic data for manganese in rodents and nonhuman primates [5]. All test reports and correspondence related to the Alternative Tier 2 Testing for MMT can be found in the Federal Docket Management System (FDMS) at http://www.regulations.gov/ identified by docket number EPA-HQ-OAR-2004-0074.

One objective of the MMT Alternative Tier 2 program was to generate data to support the development of PBPK models for manganese [5, 6]. Development of these models represents an effort that spans more than a decade. Key pharmacokinetic data needed to support PBPK model development and a paradigm for a tissue-dose-based health risk assessment for manganese were initially described by Andersen and coworkers [7] in 1999 and helped guide future studies. Numerous animal experiments have subsequently addressed many of the data gaps raised by Andersen and coworkers [7] (reviewed in [5, 6, 8]). This manuscript describes the development of a series of PBPK models for manganese. Moreover, we provide a framework for their application to risk assessment.

## 2. Manganese PBPK Models: Development and Status

The development of the PBPK models proceeded in a step-wise, iterative fashion with increasing model complexity being added at each step.  Table 1 provides an overview of the initial “first generation” models developed for this research program. The earliest dosimetry models were adapted from pharmacokinetic models developed for zinc, copper, and other essential metals that focused on dietary intake and deficiency. Features of these models that were deemed important for manganese include features of these models that were deemed important for manganese include the ability to describe homeostatic control of an essential element under normal and deficient dietary conditions, and the use of compartmental and linear exchange rates to distribute the essential element into tissues and cellular compartments. The earliest manganese models were used to quantitatively test assumptions regarding the movement of manganese from the rodent gastrointestinal tract (GIT) and liver [9] and to ascertain the degree to which systemic and orally derived manganese are handled similarly in the liver [10]. The resulting pharmacokinetic models accurately described the decreased gastrointestinal (GI) manganese uptake and increased hepatobiliary elimination that is seen with rising levels of manganese in the diet.

Early efforts also developed an initial framework for a multicompartment PBPK model. These models evaluated the kinetic behaviors of manganese in the brain, liver, and respiratory tract during and after manganese inhalation [12, 13]. Several model structures were considered during this developmental phase (Table 1). Ultimately, manganese kinetics were best described using a model that included dose-dependent saturable tissue binding as well as free and bound manganese [13]. In this context, bound manganese was confined to tissues and reflected basal manganese concentrations. Free manganese circulates in the blood and increasing concentrations resulted during manganese inhalation. Free manganese was rapidly cleared following exposure, thereby returning tissue manganese concentrations to their original basal levels. This rise of free brain manganese concentration was described with diffusion rate constants (*k*_in_ and *k*_out_). Peak tissue manganese concentrations were constrained by the tissue maximal binding capacity (*B*_max⁡_). Importantly, dose dependencies predicted by the Nong model [13] were consistent with the total manganese tissue levels measured in rats following manganese inhalation. The model also replicated the rapid increases in tissue manganese concentrations seen at the highest inhaled manganese concentrations, as well as the rapid return to baseline after exposure ceased. The model developed by Nong and coworkers [13] for the adult rat incorporated these and other features and was used as the basis for all subsequent “second generation” animal PBPK models (Table 2).

**Figure 1:**
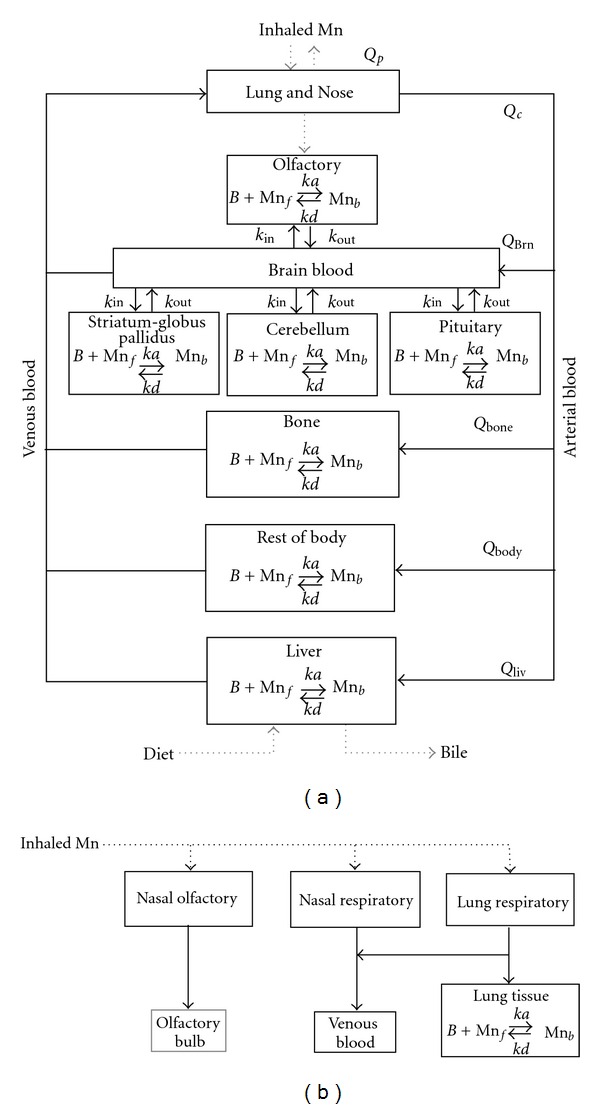
The PBPK model structure developed by Nong and coworkers [14] describing tissue manganese kinetics in adult rats. The overall PBPK model structure is shown in (a); an expanded view of the respiratory tract modeling is shown in (b). Inhaled manganese is absorbed through deposition of particles on the nasal and lung epithelium. Most of the manganese deposited in the nasal cavity is absorbed into the systemic blood while a small fraction undergoes direct delivery to the olfactory bulb. Every tissue has a binding capacity, *B*_max⁡_, with affinity defined by association and dissociation rate constants (*k*_*a*_, *k*_*d*_). Free manganese moves in the blood throughout the body and is stored in each tissue as bound manganese. Influx and efflux diffusion rate constants (*k*_in_, *k*_out_) allow for differential increases in manganese levels for different tissues. *Q*_*p*_, *Q*_*c*_, *Q*_tissue_ refer to pulmonary ventilation, cardiac output, and tissue blood flows. Reprinted from [14] (with permission).

Starting in 2009, the focus of the modeling effort began to shift to the development of more complete PBPK models for animals (Table 2). These models retained many of the features found in the Nong model [13], including dose-dependent saturable tissue capacities and asymmetrical diffusional flux of manganese into various tissue compartments. The second generation models also used airway deposition models based on particulate aerodynamics to describe manganese delivery to the respiratory tract [17]. Descriptions of the upper airways were broadened to include descriptions of the nasal cavity and olfactory epithelium using data published by Schroeter et al. [18]. Regarding the CNS, separate compartments for the olfactory bulb, striatum, pituitary gland, and cerebellum were developed. Specific influx and efflux diffusion rate constants (*k*_in_, *k*_out_) and binding constants (*B*_max⁡_, *k*_*a*_, *k*_*d*_) for different brain regions were used to account for the differential increases in regional brain manganese concentrations seen under various experimental conditions. These modifications led to the publication of the revised adult rat model depicted in Figure 1 [14]. Additional models were subsequently developed to describe lactational [15] and gestational [16] transfer of manganese in rats. In all cases, model output was compared to inhalation data obtained under this test program and that from the available literature.

**Figure 2:**
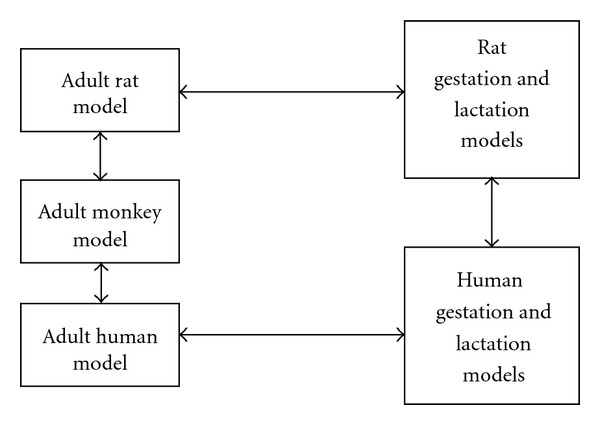
Parallelogram approach for developing Mn PBPK models for adult humans, as well as gestation and lactation.

In 2009, Nong and coworkers also described the development of a PBPK model for nonhuman primates from the revised adult rat model [14]. The monkey PBPK model was viewed as a critical step in the evolution of appropriate human models (Figure 2). One goal of the modeling effort was to retain as many features present in the rat model as possible. Body weight, tissue volumes, olfactory and respiratory tissues surface areas, ventilation rates, blood flows, and certain other model parameters were adjusted to describe monkey physiology while others (biliary clearance and brain diffusional fluxes) were allometrically scaled based on body weight. Tissue-specific binding capacities were scaled to their respective tissue volumes while tissue-binding rate constants (*k*_*a*_ and *k*_*d*_) were nearly constant from rat to monkeys. Dietary uptake and basal biliary excretion rates were also adjusted to fit measured background tissue manganese concentrations.

The final steps in the modeling program were to develop PBPK models for humans (Table 3). The starting point for this effort was the monkey PBPK model developed by Nong et al. [14] with appropriate changes in physiological descriptions, allometric scaling of biliary clearance and brain diffusional fluxes to body weight, and small changes in tissue binding rate constants (*k*_*a*_ and *k*_*d*_). A significant change in the model involved the use of a more physiological description of the GIT to address an apparent delay in GI absorption evident in tracer Mn studies in primates [19] and the differential enterocyte turnover rates across lifestages [20]. Schroeter and coworkers [19] included a series of gut compartments (e.g., GI lumen and epithelium) to better describe the absorption of ingested manganese and storage of this metal. The epithelial linings of the small and large intestine have a high cellular turnover and contain rapidly proliferating cells (enterocytes) which replace those that are shed into the lumen. Enterocytes are an important site for metal uptake and ultimately excretion through the sloughing of these cells. In our model, manganese transfer from the upper GIT epithelium to the lower GIT resulted from sloughing of enterocytes from the epithelial layer. The manganese found in shed enterocytes was ultimately excreted into feces without entering the systemic circulation. This allowed for the differential rates of enterocyte sloughing found in different life stages to be accounted for [19, 20]. The fraction of manganese absorbed by the GIT (*F*_dietup_) and the biliary excretion rate constant (*k*_bile*C*_) were calibrated based on steady-state tissue concentrations and ^54^Mn tracer studies. Induction of biliary elimination of manganese was also included in the model. These changes in model structure were sufficient to capture the observed dose-dependent changes in manganese absorption by the GIT and biliary excretion by the hepatobiliary system. Schroeter and coworkers [19] used a step-wise approach to model development by first developing a revised monkey PBPK model, followed by an adult human model, which was validated by the available human Mn tracer data [19]. The final step in the modeling efforts culminated in the development of a model that described gestational and lactational transfer of manganese in humans [20].

## 3. PBPK Models in Manganese Risk Assessment: Why Tissue Dose Matters

As an essential metal, manganese is found in all mammalian tissues. Several homeostatic mechanisms have evolved to tightly regulate these tissue manganese concentrations within a normal range of values. For most tissues, normal manganese concentrations in humans range from 0.15 to 4 **μ**g Mn/g of wet tissue [1]. As noted earlier, manganese neurotoxicity occurs when manganese intake exceeds elimination, resulting in manganese accumulation in brain regions including the globus pallidus, which is particularly sensitive to manganese accumulation during overexposure. Although manganese neurotoxicity is sensitive to exposure dose, it is relatively insensitive to route of exposure, as similar neurological responses have been linked to prolonged high-dose manganese inhalation, drinking water ingestion, long-term total parenteral nutrition (TPN), or impaired manganese clearance because of hepatobiliary dysfunction [24]. Because of the ubiquitous nature of manganese and the role of dietary manganese in establishing steady-state tissue concentrations, risk assessments of inhaled manganese should consider the essentiality of manganese from diet to establish the tissue concentrations that will be altered with increasing levels of inhaled or ingested manganese. Therefore, to understand the risk to humans from excessive manganese exposure, it is important to determine the exposure conditions that result in manganese concentrations in the brain that are increased significantly compared with brain manganese concentrations arising from normal dietary intake [7]. Pharmacokinetic models can be used to help establish safe exposure levels by predicting exposure conditions that lead to toxicologically significant increases in tissue manganese.

## 4. Application of PBPK Models in Human Health Risk Assessment

**Figure 3:**
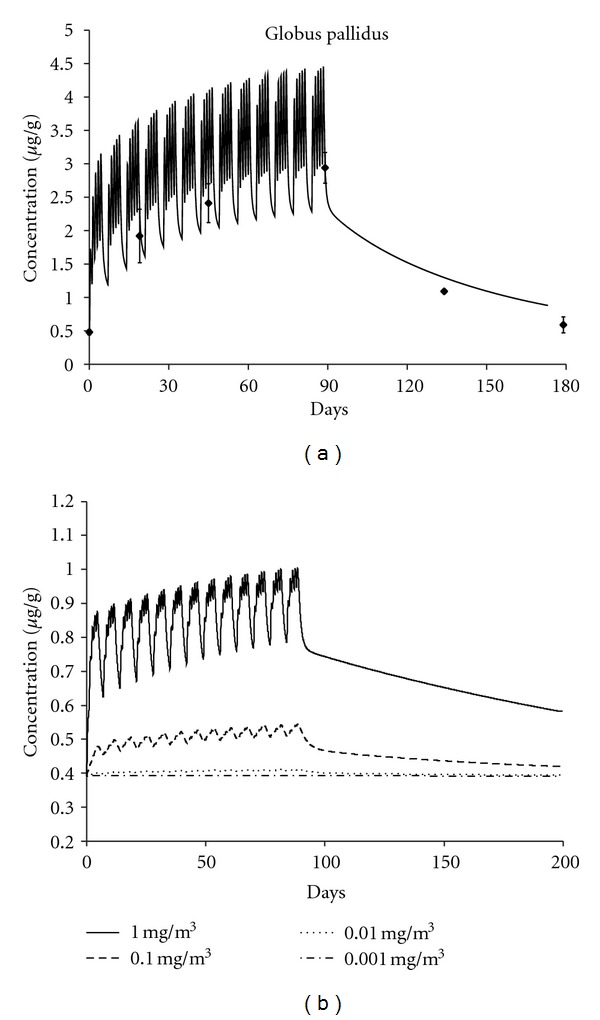
Curves showing simulated end-of-exposure brain tissue manganese concentrations in monkeys (a) and people (b) as a function of inhalation exposure concentration (mg Mn/m^3^). Simulated exposures are for 90 days (5 days/week) for either 6 h/day (monkeys) or 8 h/day (human beings). The monkey simulation results at 1.5 mg/m^3^ (a) are compared with data from Dorman et al. [21] depicted with symbols showing means and standard errors (SEs) from four to six monkeys per time-point. The larger magnitude changes predicted in monkeys compared with humans at higher inhalation exposure concentrations could be due to the saturation of manganese binding sites in the monkey coming from higher manganese concentrations in the diet of the monkeys. Modified from [19].

One of the first attempts at applying PBPK models in scenarios relevant to human health risk assessment was performed by Schroeter and colleagues [19]. These investigators used their PBPK model to predict brain manganese concentrations in monkeys and people following subchronic manganese inhalation (Figure 3). The predicted globus pallidus manganese concentrations for monkeys (Figure 3(a)) compared favorably with those observed by Dorman et al. [21] in monkeys subchronically exposed to manganese sulfate (MnSO_4_), giving added confidence that the PBPK models were designed and parameterized appropriately. The human simulations performed by Schroeter mimicked an 8 hr/day 5 day/week occupational exposure. The larger magnitude changes predicted in monkeys compared with humans at higher inhalation exposure concentrations may be due to saturation of manganese binding sites in the monkey at the higher manganese concentrations in the diet. Human diets are typically low in manganese content compared to diets in laboratory animal chows, which are often supplemented to much higher (*~*100 ppm) levels. At the lowest human exposure concentration used in our simulations (0.001 mg Mn/m^3^), the model predicted no appreciable increase (<1% change from basal concentrations) in human globus pallidus manganese concentrations above the background levels associated with normal dietary exposure (Figure 3(b)). At an exposure concentration of 0.01 mg Mn/m^3^, slight increases (*∼*5%) in globus pallidus manganese concentration above background levels were predicted during the inhalation exposure period. More significant (>30%) increases in globus pallidus manganese concentrations were predicted at the higher exposure concentrations (>0.1 mg Mn/m^3^). These data are consistent with derivations of benchmark concentrations for subclinical neurological effects from occupational studies at concentrations of 0.2 mg Mn/m^3^ [25] and indicate that significant increases in tissue manganese concentration above normal background variability are required for subclinical effects to be manifested.

In light of our success in describing the rat and monkey tissue data and concordance with human responses and specific exposures, we conducted additional simulations using the available PBPK manganese models identified in Tables 2 and 3 to address other exposure scenarios of concern to toxicologists and risk assessors. Our goal was to predict tissue concentrations in individuals with altered physiology due to developmental life stage (Scenario 1) or disease (Scenario 2). A second goal was to use the PBPK models to predict brain manganese concentrations with prolonged inhalation exposure and variable dietary manganese intake (Scenario 3). The results of these simulations could help guide risk assessors in the application of intra- or interspecies uncertainty factors (UFs) as they establish exposure guidelines for the general public (e.g., an inhalation reference concentration or RfC) or workers (e.g., threshold limit value or TLV). In most risk assessments, UFs are applied to lower the acceptable air concentration to protect potentially susceptible subpopulations or account for species differences in response. For example, the current US EPA manganese RfC derivation incorporates a composite UF of 1000 that included UFs of 10 to protect sensitive individuals, 10 for use of a LOAEL, and 10 for database limitations, such as less than chronic periods of exposure, inadequate information regarding developmental and reproductive toxicity, and uncertainty about the toxicity of various forms of manganese [26].

The alternative PBPK model-based approach we present in this manuscript results in the development of pharmacokinetic chemical-specific adjustment factors (CSAFs) (or data-derived extrapolation factors (DDEFs)) that could be used in lieu of default UFs used in most risk assessments [27, 28]. A pharmacokinetic CSAF is a ratio in humans or animals of a measurable metric for internal exposure to the active compound such as area under the curve (AUC) (AUC is a surrogate for the daily and/or cumulative manganese dose received by an individual), *C*_max⁡_, or clearance between a baseline and potentially susceptible subpopulation [27]. While serving the same purpose as UFs, these extrapolation factors are based on data directly pertinent to the chemical of interest, rather than having their basis on default assumptions about inter- and intraspecies variability [28]. This approach leads to a higher confidence in the calculated adjustment factor and contributes to consistency in regulatory processes and decisions [28]. Unless otherwise noted, all simulations in these scenarios provide results for total tissue manganese concentration.

**Scenario 1. Consideration of Potentially Susceptible Subpopulations Based on Lifestage and Pregnancy Status**

Age-related changes in physiology can influence the pharmacokinetics of xenobiotics, and some experimental data suggest that that the aged nervous system may be at increased risk following exposure to manganese. For example, manganese-induced depletion of striatal glutathione is more severe in aged (20 months old) rats than in young (3-month-old) rats following repeated (7 day) high-dose (15–100 mg Mn/kg/day) oral exposure to manganese chloride [29]. Occupational and environmental exposure studies indicate that increased age may be a risk factor for manganese-induced neurobehavioral deficits [30]. To explore this question more quantitatively, we used the rodent PBPK model, as data were available in the literature regarding the degree to which pulmonary function declines with age.

**Figure 4:**
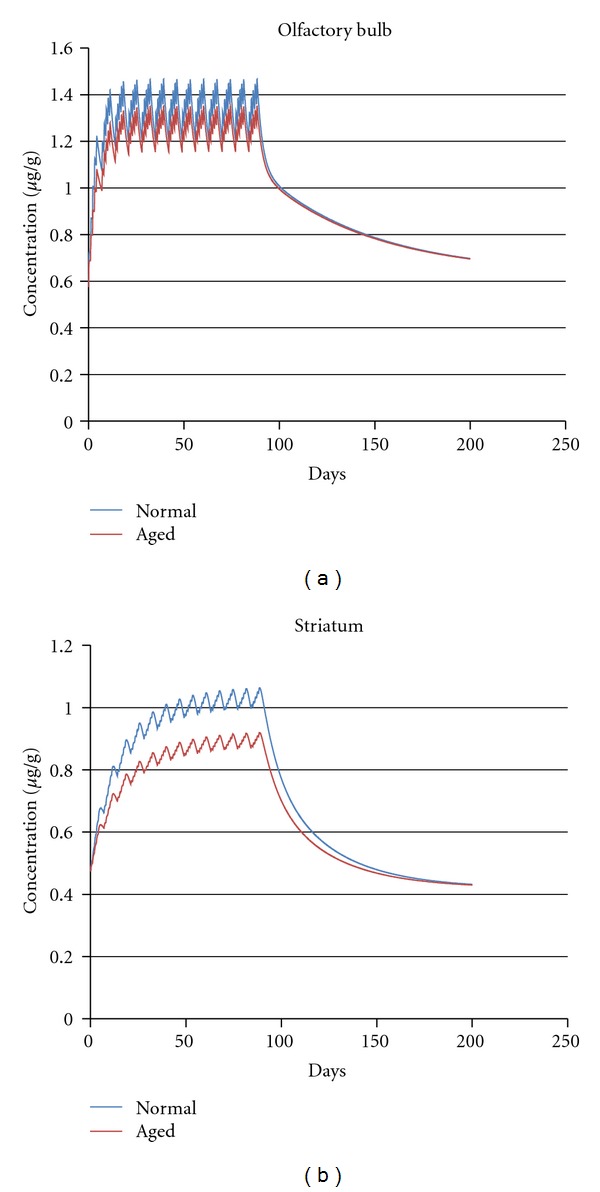
Simulated olfactory bulb (L) and striatum (R) manganese concentrations in adult and aged (16 month old) male rats following 6 hr/d inhalation MnSO_4_ exposure at 0.5 mg Mn/m^3^ for 90 days. Model simulations for aged rats had a 25% decrease in minute volume consistent with reported reduction in pulmonary function [22, 23].

Model simulations for aged rats (Figure 4) used a 25% decrease in minute volume consistent with reported reduction in pulmonary function in aged rats [22, 23]. Aged rats had lower target brain tissue manganese concentrations than middle-aged animals at the same exposures. This difference is likely due to the decreased breathing rates and pulmonary capacity of aged animals [31]. Since the manganese tissue concentration in the potentially susceptible subpopulation was less than in adult males, a pharmacokinetic CSAF for the aged life stage should be <1. Extending this simulation from rats to humans will require some changes to the physiological parameters used in the current models. For example, changes in liver volume and hepatic blood flow that significantly impact xenobiotic metabolism and clearance occur in geriatric human patients [32]. Likewise, aged humans have altered biliary [33], pulmonary [34], and gastrointestinal [35] function that may alter manganese pharmacokinetics. These complexities require a human model simulation of this scenario that lies outside of the focus of the present manuscript. However, we have provided the template for generating a pharmacokinetic CSAF for aged individuals. This preliminary analysis indicates that large adjustment factors may not be necessary to extrapolate from adult males, which typically are the subject of the occupational epidemiological studies that determine the point of departure in manganese risk assessments, to aged life stages.

Evaluation of other life stages is also important since they are also covered in the extrapolations from occupational studies by UFs. Epidemiological studies in children have reported an association between elevated dietary manganese exposure and neurobehavioral and neurocognitive deficits [36]. Concerns for potential vulnerability to manganese neurotoxicity during fetal and neonatal development have been raised by regulatory agencies [37]. PBPK models developed for this program can be used to quantitatively estimate the role that higher intestinal absorption of ingested manganese and a lower basal biliary excretion rate seen in children (see [1] for review) affect dose-to-target-tissue during manganese inhalation. To this end, Yoon and coworkers [20] used their human PBPK model to estimate average expected daily AUCs for the globus pallidus among different life stages (nonpregnant, pregnant, and lactating women, fetuses, nursing infants, and 3-year-old children) for three different exposure conditions, namely, 0, 0.001, and 0.01 mg Mn/m^3^ for 24 hr/day 7 day/week (see [20], Figure 7). Exposure durations in the different simulations varied with life stage (~9 month pregnancy for women and their fetuses; 4 months for nursing infants and their mother, and 3 years after birth for children). The average daily AUC to the globus pallidus was calculated at the end of each simulation after running the model for appropriate durations for the selected life stages. Pregnancy and lactation did not greatly affect the simulated internal dose of Mn in the brain. The average daily AUCs were greater in men than in women at high-dose environmental exposure of Mn, that is, exposures where brain Mn concentration started to rise from the basal level. The change in the average daily globus pallidus AUC resulting from high-dose manganese inhalation was greater in nursing infants compared to other life stages due to the lower (marginally deficient) basal levels of tissue manganese at this age [20]. For example, the estimated average AUC in the highly exposed infants was approximately 11.5 *μ*g Mn*·*hr/g/day compared to approximately 7.9 *μ*g Mn*·*hr/g/day in unexposed infants, while the AUC in the adult males increased from 9.3 *μ*g Mn*·*hr/g/day to 11.5 *μ*g Mn*·*hr/g/day. This apparent increase in sensitivity toward increasing tissue concentration is due to the lower basal tissue levels in neonates and likely represents the enhanced demand for manganese during this life stage [20, 38]. Both in adults and children after weaning, the relative contribution of inhalation (0.01 mg/m^3^) was smaller than their normal dietary intake at these inhalation exposures. Because no life-stage achieved a higher AUC than adult males, the PK simulations support a pharmacokinetic CSAF of 1 for fetal, neonatal, pregnant, and nonpregnant female life-stages.

**Scenario 2. Consideration of Individuals with Moderate-to-Severe Hepatobiliary Dysfunction**

Individuals with severe hepatobiliary disease often develop elevated brain manganese concentrations when compared with normal individuals [39]. For example, Rose and coworkers [39] reported brain manganese concentrations in autopsy samples taken from patients with liver cirrhosis. When compared to age-matched controls, cirrhotic patients had significantly elevated brain manganese concentrations. These increases were most marked in the globus pallidus, where patients with severe cirrhosis had mean (±SEM) pallidal manganese concentrations of 4.04 ± 1.54 *μ*g/g versus 1.41 ± 0.91 *μ*g/g in controls. Rose and coworkers [39] also examined whether rat models using an end-to-side portacaval anastomosis or an intracholedochal injection of formalin and concomitant ligation of the bile duct to create an experimental model of biliary cirrhosis could mimic these findings. As expected, rats with biliary cirrhosis or a portacaval-shunt had increased pallidal (increased by 27% to 57%) and caudate/putamen (increased by 57 to 67%) manganese concentrations when compared with sham operated or normal control groups. It is important to note that the humans and animals studied by Rose had significant clinical liver disease. For example, the cirrhotic patients studied by Rose et al. [39] died of an extreme form of hepatic encephalopathy (i.e., hepatic coma).

Individuals with liver or biliary cirrhosis can also develop significant changes in hepatic blood flow that may affect manganese pharmacokinetics. Annet and coworkers [40] reported that patients with chronic liver disease of various grades (i.e., Child-Pugh classes A, B, and C) had mean (±SD) apparent liver perfusion rates of 36.29 ± 17.96 mL/min/100 mL liver volume versus 65.22 ± 24.73 mL/min/100 mL liver volume in patients without liver cirrhosis (as measured using magnetic resonance imaging). An approximately 50% reduction in total hepatic blood flow has been seen in dogs with side-to-side or end-to-side portacaval anastomoses [41]. Other investigators have reported significant (*∼*50%) reductions in hepatic blood flow in rats with common bile duct ligation [42]. A similar observation is also seen in people with hepatic cirrhosis [43]. These studies indicate that there should be a 50% reduction in hepatic blood flow in the PBPK model parameters for consistency with the observations of experimentally induced hepatobiliary dysfunction.

Impaired secretion of bile acids, bilirubin, and other organic anions, consistent with reduced biliary function, is also observed in liver disease [44]. For example, rats with mild hepatic stenosis have an approximate 15% reduction in basal bile flow when compared with control animals [45]. One animal model in which biliary function has been quantitatively examined is liver dysfunction induced by the subchronic to chronic administration of TPN, an intravenous diet given in severe cases of GI disorders. Das et al. [46] reported that rabbits receiving TPN will develop qualitatively similar decreases in bile flow (reduced by 60%), bile acid secretion (52%), and sulfobromophthalein (BSP) excretion (38%) when compared with control animals. These animals also developed hepatocellular degeneration and portal tract inflammation. Thus, the available data support using a 50% reduction in bile flow in the PBPK model simulation.

**Figure 5:**
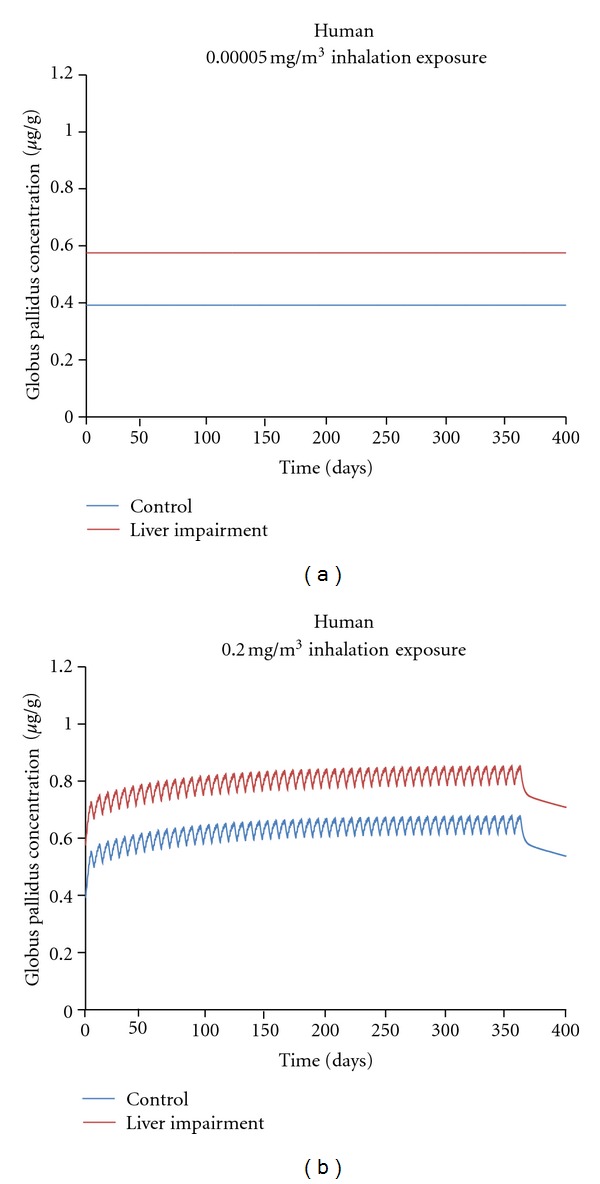
Simulated globus pallidus manganese concentrations in humans following inhalation exposure to MnSO_4_ at 0.00005 (a) or 0.2 mg (b) Mn/m^3^ for 8 hr/d, 5 d/wk, for one year. Simulations were performed using the human model developed by Schroeter et al. [19] with the following exceptions: model simulations for humans with hepatobiliary impairment had a 50% decrease in liver blood flow and a 50% decrease in biliary excretion (*K*_bile*C*_) to simulate moderate hepatobiliary disease (see text for more details).

In this study, we used the Schroeter et al. [19] model to simulate globus pallidus manganese concentrations in humans following a one year inhalation MnSO_4_ exposure to either 0.00005 (the USEPA RfC) or 0.2 mg (the current ACGIH TLV) Mn/m^3^ for 8 hr/d, 5 d/wk. Model simulations for people with hepatobiliary impairment had a 50% decrease in liver blood flow and a concomitant 50% decrease in biliary excretion (*K*_bile*C*_) consistent with changes reported in people and/or animals with liver disease as discussed above. The simulations show that, regardless of inhalation concentration, the model predicted higher pallidal brain manganese concentrations due to hepatic dysfunction alone (Figure 5), which was expected based on available data. Inhalation at the RfC had no significant effect on pallidal concentrations, regardless of hepatobiliary function (Figure 5(a)). Inhalation concentrations at the TLV produced an increase in end-of-exposure pallidal manganese concentrations that were in addition to the increase from hepatobiliary disease (approx. 0.85 *μ*g/g versus 0.68 *μ*g/g in controls, Figure 5(b)). A CSAF of ~1.25 (0.85/0.68) is supported by the PBPK modeling for this extremely sensitive subgroup. Since this CSAF value was determined at occupational exposure levels, and no changes were observed at the RfC, this disease-related CSAF is likely conservative for environmental exposures which do not cause tissue accumulation.

**Scenario 3. Consideration of Dietary Mn Variability and Chronic Manganese Inhalation**

**Figure 6:**
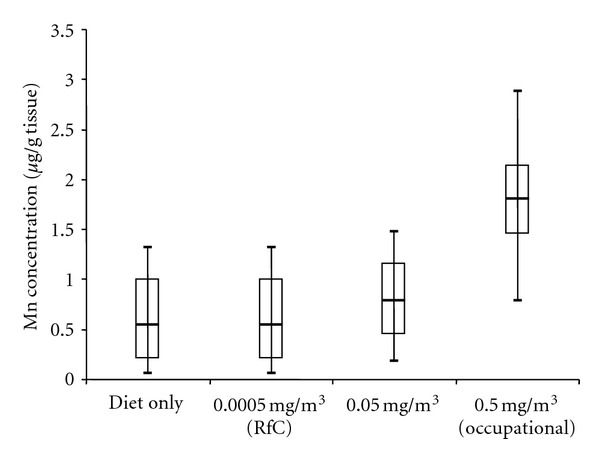
Distributions (min, 5th, med, 95th and max) of globus pallidus concentrations simulated for a human population with the input distributions described in Scenario 3 (see text for more details). Comparison of steady-state brain manganese concentration following 365 days of continuous exposure (24 hr/7 days). There is an overlap of tissue Mn levels between inhaled exposure and dietary variability. Changes in globus pallidal manganese concentrations from exposures <0.05 mg Mn/m^3^ are small when compared to the impact of normal dietary variation.

The strengths of using PBPK models in risk assessment include the ability to use the models to examine both dietary and inhaled intakes and support extrapolations from high to low doses, across routes, for different animal species, and for durations of exposure longer than those used in the studies that the models were based on [47]. To date, the most complete pharmacokinetic datasets for inhaled manganese available for PBPK model development and validation were developed for rats and monkeys using exposure durations of up to 90 exposure days (typically 6 hr/day, 5 days/week, reviewed in [5]). The manganese PBPK models described in this manuscript can be used to extrapolate beyond these exposure conditions. This scenario demonstrates this ability by predicting globus pallidus manganese concentrations in people following a continuous (24 hr/day) chronic (1 year) manganese exposure (Figure 6).

The monkey PBPK model developed by Nong et al. [14] scaled to humans was used to simulate globus pallidus manganese concentrations in people while varying the dietary intake. Normal variation of manganese concentration in globus pallidus due to the fluctuation in daily dietary exposure was simulated in an adult human population of 10,000 using Monte Carlo techniques. These simulations varied the daily dietary intake of manganese using published data (mean [±SD]: 2.43 ± 1.8 mg/day; range 0.07–6.2 mg/day) [48]. Published distribution values (mean) were used for body weight (70 kg), tissue volumes (as % body weight) for blood (8%), bone (12%), brain (2%), liver (3%), lung (1%), and the remainder of the body (0.74%) [49, 50]. Distribution values (mean) for tissue blood flow (as % cardiac output) for bone (4%), brain (11%), liver (23%), and nose (1%) were also used [49, 50]. The coefficient of variation used for body weight, tissue volume, and blood flow parameters was 0.30. Mean values for cardiac output and pulmonary ventilation were set at 13 L/hr/kg and 20 L/hr/kg, respectively. A coefficient of variation used for these parameters was 0.50. All parameter distributions were truncated by two standard deviations and statistical correlations of parameters were not included in our analysis. Air manganese concentrations ranged from current USEPA RfC (0.00005 mg Mn/m^3^) to 0.5 mg Mn/m^3^, a concentration that represents potential occupational exposure levels, although with continuous exposure in this case.

**Figure 7:**
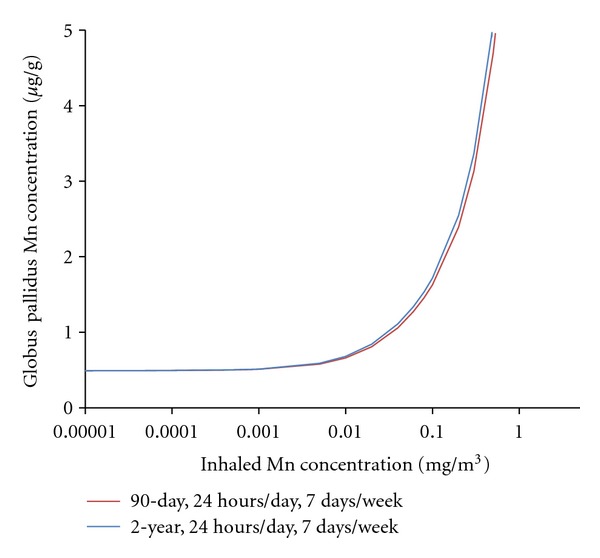
Simulated end-of-exposure nonhuman primate globus pallidus manganese concentrations following a 24 h/d, 7 d/wk inhalation for either 90 days (subchronic) or 2 yr (chronic) exposure to MnSO_4_. These simulations indicate that globus pallidus manganese concentrations are expected to rapidly reach pseudosteady-state levels during high dose manganese exposure, and that duration of exposure has a minimal effect. Its contribution only occurs once exposures reach the threshold to cause tissue accumulation.

A second question that we wanted to explore is the effect of exposure duration on the rate at which globus pallidus manganese concentrations change following manganese inhalation. Here, we compared the end-of-exposure tissue concentrations of a subchronic (90-day) versus 2-year exposure duration with the nonhuman primate model (Figure 7). Globus pallidus manganese concentrations rapidly reach pseudosteady state levels during high dose manganese exposure. These simulations are in accord with observations reported by Dorman and coworkers [21] who reported that rhesus monkeys exposed (5 d/week) for 15, 33, or 65 exposure days to MnSO_4_ at 1.5 mg Mn/m^3^ developed mean (±SEM) globus pallidus manganese concentrations of 1.92 ± 0.40, 2.41 ± 0.29, and 2.94 ± 0.23 *μ*g Mn/g tissue wet weight, respectively, all of which were significantly different (*P* < 0.05) than background tissue levels of 0.48 ± 0.04 *μ*g Mn/g tissue wet weight. Extending the simulation to 2 years produced a very slight leftward shift of the exposure-accumulation curve, but it did not change the threshold for tissue accumulation. For example, at 0.2 mg MnSO_4_/m^3^ (the current ACGIH TLV), a pharmacokinetic CSAF for subchronic to chronic duration is 1.06. Once exposure passes the threshold for tissue accumulation, a PK CSAF for duration of exposure is maximally 1.1.

Results of these simulations show several important findings. Brain manganese concentrations are controlled over a wide range of low-to-moderate exposure conditions at and above typical environmental exposures. Due to homeostatic controls, changes in globus pallidal manganese concentrations from exposures that exceed the current RfC even by several orders of magnitude (0.05 mg Mn/m^3^) are small when compared to those seen as a result of normal variation in the dietary intake of manganese as demonstrated by the Monte Carlo analysis of dietary variation (Figure 6). However, once these homeostatic mechanism(s) are overwhelmed, pallidal manganese concentrations rise rapidly. The threshold for this response appears to occur at approximately 0.001–0.01 mg Mn/m^3^ (Figure 7).

## 5. Future Applications of PBPK Models

The scenarios that we have explored here can easily be broadened to address other concerns raised in relation to the human health risk assessment of manganese. Another scenario that may prove useful to risk assessors is a consideration of the effect of altered iron homeostasis on manganese pharmacokinetics, since iron deficiency and iron-deficient anemia exist worldwide [51]. Inadequate tissue iron status resulting from dietary iron deficiency or anemia can lead to altered brain manganese deposition in animals [52–54]. It is unknown whether interactions between iron and manganese for divalent metal transporter 1 (DMT1) and other shared cellular membrane metal transporters account for this effect [55, 56]. Once these pathways and interactions are more fully elucidated, especially with quantitative measurements, these features can be incorporated into the existing PBPK models.

Although the PBPK models were originally created to support the risk assessment of combustion products of the fuel additive MMT (see [5]), they have much broader application to toxicologists and risk assessors. PBPK models can consider the impact of particle size and solubility on manganese dosimetry, especially as it relates to nanomaterials. Manganese nanoparticle exposure may occur during occupational exposure scenarios, potentially including welding. Nanoparticles display several curious inhalation pharmacokinetic behaviors that may be independent of chemical form [57]. For example, inhaled nanomaterials are deposited extensively in the nasal cavity [58]; in addition, a large percentage (~75%) of the nanomaterials that reach the alveolar region remain at that site, with less than 5% of inhaled nanoparticles translocating out of the lungs [59]; charged nanoparticles are more likely to travel to the brain via axonal transport within the olfactory nerve than are neutral nanoparticles [60]; and nanoparticle size also influences organ distribution and renal excretion [61, 62]. Several PBPK models have been developed for nanoparticles [11, 62–64]. These models were parameterized and validated using experimental pharmacokinetic data collected for different nanomaterials (e.g., iridium, silver, or technetium-labeled carbon nanoparticles) using data obtained from rats or humans. There is only sparse data on the pharmacokinetics of manganese-based nanoparticles. Some work examined translocation of manganese oxide (MnO_2_) nanoparticles from the nasal cavity to the brain [65]; however, these investigators relied on the use of nasal instillation rather than inhalation. Elder and coworkers [66] exposed rats to MnO_2_ nanoparticles with individual aerodynamic diameters of 3–8 nm (note these particles form *∼*30 nm agglomerates in the exposure system) for 6 hr/day, 5 days/wk, for a total of 12 inhalation exposure days. In this study, the olfactory bulb showed the greatest changes in proinflammatory gene expression when compared to the midbrain, striatum, and other brain regions. This finding supports the conclusion that inhaled manganese nanoparticles, like larger particles, can undergo olfactory transport from the nasal cavity to the olfactory bulb. However, PBPK modeling in rodents and MRI analysis in primates have demonstrated that the olfactory pathway does not appear to significantly impact manganese delivery to tissues outside of the olfactory pathway [67, 68]. While research on manganese nanoparticles is still limited, there is some evidence that soluble manganese may be more bioavailable and cause more effects relative to equivalent amounts of nanoparticle manganese. Whereas most of the deposited nanomaterial appears to stay in the lung, soluble manganese is readily bioavailable [14, 59, 69]. Furthermore, manganese nanoparticles appear to be less toxic than an equivalent dose of soluble Mn^2+^. Daily intratracheal instillation of soluble MnCl_2_ in rats for 3–6 weeks led to increased brain manganese levels, a reduction in body weight gain, and a decrease of open field motility when compared to controls, whereas the equivalent dose of MnO_2_ nanoparticles had no significant effects [65]. Also, MnO_2_ nanoparticles were less toxic to PC-12 cells *in vitro* by the MTT assay than an equivalent dose (in ug/mL) of soluble Mn acetate [70]. Thus, the current PBPK modeling, based on soluble manganese (MnSO_4_), may represent a worst-case scenario relative to nano-manganese after accounting for differences in pulmonary deposition. This expectation will be further clarified as more data become available comparing the pharmacokinetics and pharmacodynamics of nano- and soluble manganese.

Another potential application of the manganese PBPK models for risk assessment is the evaluation of the literature on the neurological outcomes of manganese exposure in primates, potentially identifying tissue concentrations that lead to adverse effects. This assessment would allow the models to explore the pharmacodynamic aspects of Mn exposure. The models could then be used to select the most appropriate dose metric for establishing a point of departure in future risk assessments. A previous attempt to evaluate dose response for the effects of Mn in experimental animals [71] relied on estimated cumulative intake of Mn as the only measure for comparison across studies with different doses, durations, and exposure routes. Alternative toxicologically relevant dose metrics, including estimated peak concentration, average concentration, and cumulative dose (i.e., AUC) during the Mn exposure period could be estimated using a PBPK model known to accurately account for dose dependencies of Mn distribution in the monkey for combined inhalation and dietary exposures. A large nonhuman primate response literature exists for analysis, including exposures by inhalation, oral, intraperitoneal, and subcutaneous dose routes, and spanned durations up to 2 years [71]. This type of analysis using PBPK models is currently underway and will make it possible to provide a consistent description of the dose response relationship for the effects of Mn independent of exposure route.

Finally, PBPK models may be used in an alternative dosimetric-based risk assessment strategy for essential elements considering dietary intake, natural tissue background levels, and dietary and population variability. An upper safe exposure value could be based on an air concentration that changes brain tissue levels by no more than some fraction of the normal variability within a healthy population [7, 72]. The relationship between exposure levels and target-tissue levels would be determined by the use of PBPK models, which would account for the existence of the dose-dependent transition (i.e., threshold level) for accumulation. This methodology is inherently conservative with respect to neurological outcome, as the air guideline would be set to prevent only tissue accumulation. Potentially sensitive subpopulations as described in the scenarios here can be quantitatively taken into account with PBPK modeling instead of the application of UFs as described in the scenarios here. Another key advantage of a pharmacokinetic approach for risk assessment is that it is not reliant upon existing occupational studies, which have limitations with respect to exposure assessment, evaluation of adverse effects, and establishing causation (reviewed in [30]), to establish a point of departure [72]. Similar PBPK model-based dosimetry approaches should also be considered for risk assessments with other essential metals, such as copper and zinc. However, the development of a comprehensive PBPK model for any essential element depends on the availability of a sufficiently diverse and robust data set to enable model construction and validation. These data now exist for Mn, due in large part to the Alternative Tier 2 testing program for MMT [5].

## 6. Conclusions

The development of PBPK models facilitates more rigorous quantitative analyses of the available pharmacokinetic data and allows the comparison and consideration of dose to target tissue in risk assessment decisions. While PBPK modeling for many exogenous compounds has become routine, there are significantly more challenges in understanding the full set of biological factors that control uptake, distribution, and clearance of manganese and other essential nutrients that exert toxicity at high doses. An overarching goal of our modeling efforts was to evaluate situations that may lead to increased brain accumulation due to altered manganese regulation in healthy and potentially susceptible human populations. These subpopulations, identified as part of the Alternative Tier 2 testing program for MMT, include adult males, females, the aged, fetuses, neonates, and pregnant women, as well as those with high or low dietary manganese intake. Pharmacokinetic CSAFs were calculated to extrapolate from adult males, which are the typical subjects evaluated in occupational manganese studies, to these other life stages. These values were all ≤1, indicating that no pharmacokinetic adjustment is needed to account for these populations. Regarding duration of exposure, a CSAF of 1 to 1.1 is calculated depending on the inhalation concentration once exposure levels increase above the threshold for tissue accumulation (i.e., 0.001–0.01 ug/m^3^). In addition, while diseased individuals are not typically included in the extrapolations done in typical health risk assessments of environmental exposure, we have now extended the simulations to include those with moderate to severe hepatobiliary insufficiency to represent a population at a higher risk of manganese effects. The simulations suggest that an impaired individual may have elevated brain manganese concentrations regardless of manganese inhalation levels, and typical environmental levels do not increase this burden. At higher exposure levels, a CSAF of 1.25 is derived for the extremely sensitive subgroup. In total, the pharmacokinetic CSAFs developed here are less than the pharmacokinetic portion of the typical UF of 10 for human variability incorporated in current risk assessments of ambient manganese exposure. Future efforts can refine the scenarios presented here in humans, examine the effect of iron homeostasis, and evaluate the effects of particle size and solubility, including manganese nanoparticles.

A second outcome of these efforts is the increased confidence in the quantitative predictions of elevated manganese levels that might serve as a basis for a dosimetry-based risk assessment. An underlying assumption to this risk assessment approach is that elevated brain manganese concentration is a prerequisite for the development of manganese neurotoxicity. From a risk assessor's perspective, an upper safe exposure value could be based on an air concentration that changes brain tissue levels by no more than some fraction of the normal variability within a population [7, 72]. While some data gaps still exist regarding the biology of manganese transport and storage, the models described in this manuscript capture the main dose-dependent characteristics of manganese disposition. Using a Monte Carlo analysis to simulate population variability on target tissue manganese levels, modeling simulations indicate that air manganese concentrations of 0.001–0.01 mg/m^3^ are required to begin to influence natural background tissue concentrations in adult males, which are the most sensitive subgroup regarding manganese tissue accumulation currently examined. With the forthcoming evaluation of monkey studies of Mn toxicity using the model to assess dosimetry and with applications to human datasets, the current PBPK model should be an important component of future tissue-dose-based approaches for Mn risk assessment.

In summary, data collection efforts associated with the Alternative Tier 2 testing program for MMT over the past 10 to 15 years on tissue Mn after inhalation exposures at different dietary levels and associated PBPK modeling have greatly improved understanding of the integration of multiple processes that collectively control Mn concentrations in various tissues. These efforts produced a multidose route, multispecies PBPK model that recapitulates dose-dependent brain accumulation on excessive exposures. The key biological characteristics required in fitting the model to the rat and monkey tissue time course data were finite capacities for tissue binding, slow dissociation of bound Mn, dose-dependent elimination from liver and dose-dependent uptake from the diet. As expected with control process for an essential metal with high-exposure toxicity, the physiological processes preserve body stores of Mn at low intakes accelerate excretion and reduce oral absorption at higher intakes. These dose-dependent processes are well known in a general manner, but not in terms of every biochemical detail. Biological determinants for tissue binding, membrane transport, Mn retention in enterocyte and sloughing of these cells into the intestinal lumen are under investigation. Further detail regarding these steps will refine specific parameters in the current PBPK model. One area ripe for inclusion in the next-generation PBPK model is increased knowledge of metal transporters [73–75]. More detailed dose-dependencies for these transporters would elaborate the asymmetric, dose-dependent uptake processes into brain in the present model [76]. However, the relative rate constants cannot vary significantly from the current ones since the simulations with the current model show very good correspondence with all available tissue time-course data. Thus, despite some gaps in understanding the underlying biology, the PBPK modeling with Mn shows clearly how similar processes work to control basal Mn levels to a remarkably common concentration across species and how they accomplish control of tissue Mn in the face of widely different dietary intake. Controls for Mn after inhalation include enhanced elimination but lack the ability to restrict absorption through the lung epithelium afforded by the gut epithelium for oral ingestion of the metal. Clearly, the current human PBPK model stands poised to assist in further integration of emerging knowledge into a more quantitative and biologically complete description of regulation of Mn in the body. Future work, potentially including uncertainty and sensitivity analyses, will examine the inherent limitations of modeling and scaling to help determine the precise level of confidence that can be ascribed to the current predictions of human globus pallidus manganese concentrations. However, in its present form, the human PBPK model for Mn already provides a solid foundation for improving risk assessment for this essential metal that also causes neurotoxicity in humans with higher dose exposures.

## Figures and Tables

**Table 1:** Overview of initial “first generation” pharmacokinetic models developed for manganese.

Model goal(s)	Brief model description	Route(s) of exposure^‡^ and species	Mn pharmacokinetic data set(s) used in model development	Reference
Describe dose dependent gastrointestinal uptake and biliary elimination of Mn	Two-compartment distribution model that described Mn movement between the intestinal lumen and the liver using simple rate constants (*k*_in_ and *k*_out_).	Mn: O, INH ^54^Mn: IV Rodent	Tracer studies evaluating ^54^Mn whole-body elimination kinetics including a dietary Mn balance study, two biliary elimination studies, and one acute and one chronic study.	[9]
Develop quantitative descriptions of Mn delivered to the liver from the systemic circulation.	Gut lumen, liver blood, systemic blood, and a tissue compartments. Model parameters described gut uptake, ^54^Mn tracer kinetics, and hepatic extraction of Mn from oral and systemic pools.	Mn: O, INH ^54^Mn: IV Rodent	Animals exposed to either inhaled or dietary Mn. These studies also evaluated ^54^Mn whole-body elimination kinetics.	[10]
Describe the olfactory transport of Mn.	Compartments included: blood, olfactory epithelium, olfactory bulb, olfactory tract and tubercle, and striatum. Each compartment included a free and bound fraction.	^54^Mn: INH Rat	Rats exposed (90 min) nose-only to either exposure to ^54^MnCl_2_ or ^54^MnHPO_4_.	[11]
Develop the basic structure of a multiroute PBPK model for Mn.	Blood, brain, respiratory tract (nasal and lung), liver, kidneys, bone, and muscle (rest of body) compartments consisting of a “shallow” tissue pool in rapid equilibration with blood and a “deep” tissue store, connected to the shallow pool by transfer rate constants [1].	^54^Mn: IP, INH Rodent	Rodent tracer studies describing ^54^Mn distribution to various tissues and ^54^Mn elimination kinetics.	[12]
Develop a multiroute Mn PBPK model for adult rats.	Same compartments as above [4]. Model A used simple rate constants [1] to describe inter-compartmental movement of Mn. Model B had tissue binding kinetics described by dissociation and association constants (*k*_*d*_ and *k*_*a*_), and maximum concentration of binding capacity (*B*_max⁡_).	Mn: O, INH ^54^Mn: IV Rat	Rats fed on diets containing 2 to 100 ppm Mn, Rats fed a diet containing 125 ppm Mn and exposed via inhalation at 0.0 to 3.00 mg Mn/m^3^ each day for 14 d. Rats exposed to 0.1 or 0.5 mg Mn/m^3^ for 6 h/d, 5 d/wk over a 90-day period.	[13]

^‡^O: oral; IP: intraperitoneal; IV: intravenous; INH: inhalation. Where applicable, Mn tracer form and route of exposure have also been provided.

**Table 2:** Overview of “second generation” PBPK models developed for manganese.

Model goal(s)	Brief model description	Route(s) of exposure^‡^ and species	Mn pharmacokinetic data set(s) used in model development	Reference
Develop a multiroute Mn PBPK model for adult rats and monkeys.	Blood, brain (striatum, pituitary gland, olfactory bulb, and cerebellum), respiratory tract (olfactory mucosa and lung epithelium), liver, kidneys, bone, and “rest of body” compartments. Saturable Mn binding in all tissues, preferential accumulation of Mn in several brain regions. Deposition of Mn within the respiratory tract and olfactory uptake and “nose-to-brain” Mn transport were based in part on additional models describing regional particle deposition within the respiratory tract.	Mn: O, INH Rat Rhesus monkey	Rat 14- and 90-day inhalation studies. In monkeys, model parameters were first calibrated using steady-state tissue Mn concentrations from rhesus monkeys fed a diet containing 133 ppm Mn. The model was then applied to simulate 65 exposure days of weekly (6 h/day; 5 days/week) inhalation exposures to soluble MnSO_4_ at 0.03 to 1.5 mg Mn/m^3^.	[14]
Develop a PBPK model for lactating dam and neonates.	Same compartments for the dam and pups as above [6] except for excluding pituitary gland and including mammary gland (dam only). Saturable binding and other model features similar to above [6]. Dietary (e.g., transfer of free Mn in milk) and inhalation inputs to pups.	Mn: O, INH Rat	Dams and their offspring were exposed to air or MnSO_4_ (0.05, 0.5, or 1 mg Mn/m^3^) for 6 h/day, 7 days/week starting 28 days prior to breeding through postnatal day 18.	[15]
Develop a PBPK model that could predict fetal Mn dose and Mn disposition in the dam and fetus following maternal Mn exposure.	Same compartments for the dam as above [6] except for excluding the pituitary gland and including the placenta. Fetal model included blood, brain, lung, bone, liver, and “rest of body” compartments. Saturable binding and other model features similar to above [6]. Placental transfer to fetus.	Mn: O, INH Rat	Dams fed a 10-ppm Mn diet were exposed to air or MnSO_4_ (0.05, 0.5, or 1 mg Mn/m^3^) for 6 h/day, 7 days/week starting 28 days prior to breeding through gestation day 20.	[16]

^‡^O: oral; INH: inhalation.

**Table 3:** Overview of human PBPK models developed for manganese.

Model goal(s)	Brief model description	Route(s) of exposure^‡^ and species	Mn pharmacokinetic data set(s) used in model development	Reference
Refine the multi-route Mn PBPK model for monkeys and extend to human beings.	Blood, brain (globus pallidus, pituitary gland, olfactory bulb, and cerebellum), respiratory tract (olfactory mucosa and lung epithelium), liver, kidneys, bone, and “rest of body” compartments. More extensive description of gastrointestinal tract (gut lumen and epithelium) and peritoneal cavity. Saturable Mn binding in all tissues. Preferential accumulation of Mn in several brain regions. Deposition of Mn within the respiratory tract and olfactory uptake and “nose-to-brain” Mn transport were based in part on additional models describing regional particle deposition within the respiratory tract.	Mn: O, INH ^54^Mn: IV, IP, O, SC Rhesus monkey Human	Monkey inhalation study used previously [6]. Whole-body elimination or fecal excretion data available from ^54^Mn tracer kinetic studies in monkeys and people	[19]
Develop a PBPK model that could predict fetal Mn dose and Mn disposition in women and fetus following maternal Mn exposure.	Lactation and gestation models similar to those developed for rodents [15, 16]. Same compartments for women as above [9] except for excluding the pituitary gland and including the placenta and mammary gland. Fetal model included blood, brain, lung, bone, liver, and “rest of body” compartments. Saturable binding and other model features similar to above [9]. Key model features included: placental Mn transfer via active transport, lactational transfer of Mn used diffusion-mediated secretion, higher gut absorption in nursing neonates, low but inducible biliary excretion of Mn in neonates, transition of neonatal features of gut absorption and biliary excretion to those of adults, and enhanced brain uptake of Mn during fetal and postnatal development.	Mn: O, INH Human	Variety of data obtained in people including: reported brain Mn concentration at birth and children, Mn concentrations in the umbilical cord, milk, newborn blood, bone, and other tissues. Age appropriate tissue weights and blood flows.	[20]

^‡^O: oral; IP: intraperitoneal; IV: intravenous; INH: inhalation; SC: subcutaneous. Where applicable, Mn tracer form and route of exposure have also been provided.
